# Neuropsychiatric disorders, chronotype and sleep: A narrative review of GWAS findings and the application of Mendelian randomization to investigate causal relationships

**DOI:** 10.1111/gbb.12885

**Published:** 2024-02-15

**Authors:** Shane Crinion, Derek W. Morris, Lorna M. Lopez

**Affiliations:** ^1^ Centre for Neuroimaging, Cognition and Genomics, School of Biological and Chemical Sciences University of Galway Galway Ireland; ^2^ Department of Biology Maynooth University Maynooth Ireland

**Keywords:** chronotype, genome‐wide association studies, Mendelian randomization, neuropsychiatric disorders, sleep

## Abstract

Genome‐wide association studies (GWAS) have been important for characterizing the genetic component and enhancing our understanding of the biological aetiology of both neuropsychiatric disorders and sleep‐related phenotypes such as chronotype, which is our preference for morning or evening time. Mendelian randomization (MR) is a post‐GWAS analysis that is used to infer causal relationships between potential risk factors and outcomes. MR uses genetic variants as instrumental variants for exposures to study the effect on outcomes. This review details the main results from GWAS of neuropsychiatric disorders and sleep‐related phenotypes, and the application of MR to investigate their bidirectional relationship. The main results from MR studies of neuropsychiatric disorders and sleep‐related phenotypes are summarized. These MR studies have identified 37 causal relationships between neuropsychiatric disorders and sleep‐related phenotypes. MR studies identified evidence of a causal role for five neuropsychiatric disorders and symptoms (attention deficit hyperactivity disorder, bipolar disorder, depressive symptoms, major depressive disorder and schizophrenia) on sleep‐related phenotypes and evidence of a causal role for five sleep‐related phenotypes (daytime napping, insomnia, morning person, long sleep duration and sleep duration) on risk for neuropsychiatric disorders. These MR results show a bidirectional relationship between neuropsychiatric disorders and sleep‐related phenotypes and identify potential risk factors for follow‐up studies.

## Graphical abstract

Mendelian randomization (MR) uses genetic variants as instrumental variants for exposures [e.g., chronotype (preference for morning or evening time)] to investigate their causal relationship with an outcome [e.g., schizophrenia (SCZ), under a number of assumptions]. This review of MR studies summarises the bidirectional relationship between neuropsychiatric disorders and sleep‐related phenotypes.

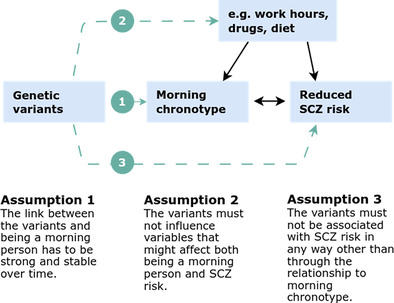

## 1 INTRODUCTION

Neuropsychiatric disorders are a complex group of disorders affecting brain function, behaviour and cognition.^1^ The major disorders include the adolescent and adult‐onset disorders of bipolar disorder (BD), major depressive disorder (MDD) and schizophrenia (SZ), and the childhood‐onset conditions of autism spectrum disorder (ASD) and attention deficit hyperactivity disorder (ADHD). These disorders were the original disorders identified for gene discovery research by the Psychiatric Genomics Consortium (PGC).^2^

There is wide variability in the characteristics of neuropsychiatric disorders which makes the identification of environmental and genetic risk factors difficult. The diversity in cognitive profile and behavioural characteristics between BD, MDD and SZ^3^ suggests the presence of a neurodevelopmental continuum with variations in cognitive impairment, negative symptoms, positive symptoms and mood disturbance.^4^ ASD and ADHD have an earlier clinical onset and a wide range of clinical features, including global or local impairment of social skills, learning and executive functions.^3^, ^4^

Circadian rhythms are patterns in physiology and behaviour that recur with 24 h frequency and are driven by endogenous mechanisms and entrained to environmental time cues.^5^ Overall regulation of the human circadian rhythms is maintained by a central clock located in the suprachiasmatic nuclei (SCN), a pair of small nuclei containing 20,000 neurons located in the anterior hypothalamus. The SCN coordinates or ‘orchestrates’ circadian rhythms at a systemic level through neural and endocrine mechanisms. The molecular clock is the core element of circadian rhythms that is present in virtually every cell, and that can even be reproduced in vitro in cell culture. The molecular clock confers 24 h rhythmicity on physiology and behaviour, but endogenous regulation of most of these rhythms will fail in the absence of SCN input. Circadian rhythm alterations can be assessed by SCN‐regulated responses such as body heat temperature,^6^ sleep–wake activity and self‐reported measures such as chronotype.^7^, ^8^, ^9^

Chronotype is thought to be a behavioural indicator of an individual's circadian rhythm for example, being a morning person (morningness) means having a preference for sleep and activities earlier in the 24 h day, while evening chronotypes prefer later sleep, wake and activity time.^7^ Chronotype is a sleep‐related phenotype that has been used to characterize the function of the human circadian clock. Chronotype is influenced by age, biological sex and environmental factors, but it also has a strong genetic component and is one of the sleep‐related phenotypes with the largest genetic studies available to investigate its biology.^7^ Eveningness, describing someone's propensity for waking and sleeping later, has been linked to increased risk for neuropsychiatric disorders^10^ and circadian rhythm disruption,^11^ as estimated by disruption of diurnal patterns of rest‐activity such as sleep–wake cycle and core body temperature variation.^12^

Disruption of circadian timing is a feature of most neuropsychiatric disorders, manifesting as disturbed sleep and diurnal changes in behaviour.^13^ Sleep and circadian rhythm disruption is present at all stages of BD.^14^ Severe BD type I cases have showed lower circadian rhythm activity than other BD cases.^15^ MDD is up to 40% more likely in night shift workers versus day workers.^16^ Sleep abnormalities occur in up to 80% of SZ patients.^17^ Between 25%–55% of children with ADHD have sleep disturbances^18^ and 50%–80% of children with ASD have sleep problems.^19^ For comparison, sleep disturbances affect 25%–30% of adults worldwide.^10^ Disruption to overlapping biological pathways and mechanisms has also been discovered in studies on the comorbidity of circadian rhythm disruption and neuropsychiatric disorders.^20^

Life‐long disability and the severity of neuropsychiatric disorders can be arguably reduced through early intervention.^21^ However, there are currently few approaches available due to the complex and poorly understood neuropathology of neuropsychiatric disorders.^22^, ^23^ Establishing a causal relationship between chronotype and neuropsychiatric disorders via MR could identify sleep‐related phenotypes as modifiable risk factors for treating the disabling features of neuropsychiatric disorders. Further understanding of the complex relationship between neuropsychiatric disorders and environmental exposures will define new approaches for interventions.

In this narrative review, we highlight the insights gained from genetic investigations using genome‐wide association studies (GWAS) of neuropsychiatric disorders, chronotype and other sleep‐related phenotypes. We review the application of Mendelian randomization (MR) to explore the potential causal relationships between neuropsychiatric disorders and sleep‐related phenotypes. MR uses single nucleotide polymorphisms (SNPs) from GWAS as instrumental variants for exposures to study the effect on outcomes.

The approach used for this narrative review was to identify and summarize evidence for the genetic component of neuropsychiatric disorders and chronotype while also highlighting existing evidence for the interrelationship between neuropsychiatric disorders and sleep traits.

While we aimed to inform on the published studies that are available that provide a broad perspective of the genetic components of these phenotypes, we did not apply a systematic review approach. A systematic review has a specific clinical question and typically involves meta‐analysis, which was not possible here because we explored multiple phenotypes within the two categories of neuropsychiatric disorders and sleep‐related phenotypes. Although this review does not include the methodological requirements of a systematic review, this narrative review remained systematic and applied the Scale for the Assessment of Narrative Review Articles (SANRA) methodology, which outlines six items for quality assessment of narrative reviews.^24^

### 1.1 Genetics of neuropsychiatric disorders

Six neuropsychiatric disorders (ADHD, ASD, BD, Insomnia, MDD, SZ) were selected for review. GWAS have identified SNPs and led to improved understanding of the genetic risk factors for neuropsychiatric disorders and, as sample sizes continue to grow in GWAS meta‐analyses by the PGC and other groups, will likely have improved power to detect more risk variants. Therefore, we included in this review the major neuropsychiatric disorders where a large‐scale, sufficiently‐powered GWAS had been performed.

While not one of the major neuropsychiatric disorders, we also included insomnia, which affects 10% of non‐psychiatric individuals^25^ and is associated with several neuropsychiatric disorders.^3^

GWAS for neuropsychiatric disorders and accompanying downstream analyses have characterized the genetic component and showed their implicated cell types and biological processes, which are summarized in Table 1. The number of SNPs reported as genome‐wide significant loci for the selected neuropsychiatric disorders ranged from 5 (ASD) to 554 (Insomnia). Calculations of SNP based heritability (h^2^_SNP_), which represents the proportion of phenotypic variance due to all measured SNPs and can be estimated from GWAS data, ranges from 7% (insomnia) and up to 24% (SZ).

**TABLE 1:** Neuropsychiatric disorder GWAS: Main findings.

Phenotype	Cases	Controls	GWAS loci^a^	h^2^_SNP_	Enriched tissue and cell types	Enriched biological processes	Biological insights	References
ADHD	20,183	35,191	12	0.22	CNS regulatory elements.	Dopamine receptor binding. Excitatory Synapse.	LDSR: ↑ MDD, depressive symptoms; ↓ smoking and subjective well‐being.	Demontis et al^26^
ASD	18,381	27,969	5	0.12	Human neocortex modules M16 and M17. H3K4me1 histone marks. CNS. Developing brain, germinal matrix, cortex‐derived neurospheres, and ESC‐derived neurons.	Corticogenesis regulatory elements. Foetal corticogenesis.	LDSR: ↑ SZ, EA, MDD & ADHD; **↓** chronotype & subjective well‐being. PRS: Strong heterogeneity found in ASD subgroups split by diagnostic class and cognitive phenotypes.	Grove et al^27^
BD	41,917	371,549	30	0.19	Hippocampal pyramidal neurons. Interneurons of the prefrontal cortex and hippocampus. Excitatory and inhibitory neurons.	Neuronal processes. Synaptic functioning Calcium signalling Neurogenesis	LDSR: ↑ correlation with SZ, MDD, Anorexia, ADHD & ASD. Drug targets: Enrichment of psycholeptics, calcium channel blockers, antiepileptics and general anaesthetics (*HTR6, MCHR1, DCLK3* and *FURIN)*.	Mullins et al^28^
Insomnia	593,724	1,771,286	554	0.07	Tissues of cerebellar hemisphere, cerebellum, frontal cortex BA9 and anterior cingulate cortex BA24. Lateral geniculate nucleus, Habenula, ventral pallidum and anterior pretectal nucleus.	Synaptic organization, transmission and signalling. Behaviour.	LDSR: ↑ cardiovascular, metabolic and psychiatric traits. Colocalization: results indicated 2 distinct loci‐trait clusters with metabolic and psychiatric traits.	Watanabe et al^29^
MDD	246,363	561,190	102	0.09	CNS. Anterior cingulate cortex, frontal cortex and cortex brain regions and neuron brain cells. Skeletal muscle tissues.	Behaviour, cognition and synaptic transmission. Mood modulation. Emotion processing.	LDSR: ↑ SZ, BD; ↓ college completion Drug targets: *DRD2* and *NRG1*, targets of antipsychotic and antidepressant drugs.	Howard et al^30^
SZ	69,369	236,642	294	0.24	Cortical inhibitory interneurons. Excitatory neurons from cerebral cortex and hippocampus (pyramidal and granule cells). Glutamatergic neurons in the cortex, amygdala, and hippocampus.	Neuronal excitability, development, and structure. Synaptic organisation and differentiation. Modulation of chemical transmission. Postsynaptic processes implicated in risk.	Fine‐mapping: highlights convergence of rare and common variants in 4 specific genes (*GRIN2A, SP4, STAG1* and *FAM120A)*. Drug targets: *ACE*, a target for antihypertensive drugs.	Trubetskoy et al^31^

Abbreviations: ADHD, attention deficit hyperactivity disorder; ASD, autism spectrum disorder; BD, bipolar disorder; CNS, central nervous system; EA, educational attainment; ESC, embryonic stem cell; GWAS, genome‐wide association studies; LDSR, linkage disequilibrium score regression; MDD, Major Depressive Disorder; MR, Mendelian randomisation; PRS, polygenic risk score; SZ, Schizophrenia.

^a^ Independent genome‐wide significant loci (*p* < 5 × 10^−8^).

The findings from gene set enrichment analyses (GSEA) indicate that the frontal cortex, prefrontal cortex and hippocampus play an important role in these neuropsychiatric disorders. GSEA has also showed that genes associated with a neuropsychiatric disorder are significantly overrepresented in biological processes involved in development, function and signalling of neurons and synapses. Genetic correlation analyses have showed significant positive associations between numerous neuropsychiatric disorders and cognitive, behavioural and psychiatric traits (Table 1).

### 1.2 Genetics of sleep‐related phenotypes

Table 2 summarizes the main findings from GWAS and post‐GWAS analyses of sleep‐related phenotypes. Chronotype was selected as the main phenotype to study sleep‐related phenotypes due to the availability of the largest samples for genetic studies. A GWAS of 697,828 individuals was performed and detected 351 genome‐wide significant (GWS) loci associated with self‐reported chronotype.^7^ These SNPs were found in genes that control circadian regulation, cAMP, glutamate and insulin signalling pathways.

**TABLE 2:** Chronotype and sleep GWAS: Main findings.

Phenotype	Type	Individuals	GWAS loci^a^	H^2^ SNP	Enriched tissue and cell types	Enriched biological processes	Biological insights^b^	References
Chronotype	Self‐report	697,828	351	0.14	SCN genes Circadian gene and circadian‐implicated genes synapses, axons and dendrites retinal tissue	Circadian rhythm and circadian clock pathways CNS & brain development Neurogenesis. Behavioural pathways Responses to internal & external stimuli Metabolism of cyclic nucleotides G‐protein signalling and activation. NMDA glutamate signalling pathway	Fine‐mapping: Identified 10 likely causal variants, link to insulin secretion. NMDA pathway genes *NRXN1* and *RELN* influence the risk of SZ.	Jones et al^8^ ^A^
Sleep efficiency	Measured	85,502	5	0.13	Cerebellum Hippocampus	None reported.	Fine‐mapping: likely causal variant in hippocampus expressed *PDE11A*. Overlap in *MEIS1*, linked to restless leg syndrome/insomnia.	Jones et al^8^ ^B^
No. sleep episode	Measured	85,502	21	0.22	Cerebellum	Serotonin metabolic process	*APOE* ε4 allele variant, associated with late‐onset Alzheimer's and cognitive decline—increase in allele frequency by age. *CLUAP1* variant, associated with photoreceptor maintenance.	Jones et al^8^ ^B^
L5	Measured	85,723	6	0.12	Cerebellum	None reported.	*APOE* ε4 risk allele identified. LDSR: High genetic correlation with chronotype.	Jones et al^8^ ^B^
M10	Measured	85,830	1	0.09	Cerebellum	None reported	LDSR: High genetic correlation with chronotype.	Jones et al^8^ ^B^
Sleep duration	Self‐reported	446,118	78	0.09	Cerebellum, Cortex, Hippocampus, Hypothalamus	Dopamine binding, mechanosensory behaviour, striatum development	LDSR: Negative genetic correlation with insomnia and positive genetic correlation with SZ and BD.	Dashti et al^32^
Sleep midpoint	Measured	85,502	1	0.10	Cerebellum	None reported	*APOE* ε4 risk allele identified. LDSR: High genetic correlation with chronotype.	Jones et al^8^ ^B^

Abbreviations: GWAS, genome‐wide association studies; LDSR, linkage disequilibrium score regression; L5, Midpoint of least active 5 h; M10, Midpoint of most active 10 h; SCN, suprachiasmatic nucleus.

^a^ Independent genome‐wide significant loci (*p* < 5 × 10^−8^).

^b^ ↑ represents an LDSR positive genetic correlation between phenotypes and ↓ represents an LDSR negative genetic correlation between phenotypes.

Most GWAS for sleep‐related phenotypes have used self‐reported measures; however, recent GWAS have been performed on accelerometer‐recorded sleep measurements. Jones et al published a GWAS of 85,670 individuals using accelerometer measurements that detected 47 GWS loci associated with sleep quality, sleep quantity and sleep timing (Table 2).^8^ Sleep quality was estimated using measurements for (1) sleep efficiency (sleep duration divided by the time between the start and end of the first and last nocturnal inactivity period) and (2) the number of nocturnal sleep episodes. Sleep timing was estimated using (1) midpoint of least active 5 h (L5), (2) midpoint of most active 10 h (M10) and (3) sleep midpoint (Table 2). Number of nocturnal sleep episodes was found to have the largest genetic component with SNPs accounting for 22.3% of phenotypic variance (Table 2). An additional GWAS of 446,118 individuals performed by Dashti et al^32^ identified 78 GWS loci associated with self‐reported sleep duration and found SNPs associated with pathways such as dopamine binding and neurotransmission and plasticity (Table 2).

### 1.3 Genetic relationship between neuropsychiatric disorders and sleep‐related phenotypes

There is genetic evidence of a bidirectional relationship between neuropsychiatric disorders and circadian rhythm.^33^ Disruption to overlapping biological pathways and mechanisms has been discovered in studies on the comorbidity of circadian rhythm disruption and neuropsychiatric disorders.^20^ Other evidence includes a study using animal models that induced mania‐like behaviours through mutations in the *Clock* gene^34^ and overlapping mutations in the *CLOCK* gene have been linked to both neuropsychiatric disorders and circadian rhythm disruption.^35^, ^36^ Increased sleep duration and efficiency have also been linked to *PDE11A* variants, a potential target gene for mood stabilization in neuropsychiatric disorders.^37^ *PDE11A* SNPs associated with sleep duration were previously linked to schizophrenia and migraines.^8^ Gene set analysis has also been used to identify an enrichment of genes expressed in the cerebellum for all sleep measurements (Table 2), a brain region linked to neuropsychiatric disorders that, when dysfunctional, can lead to changes in sleep–wake cycle.

**FIGURE 1:**
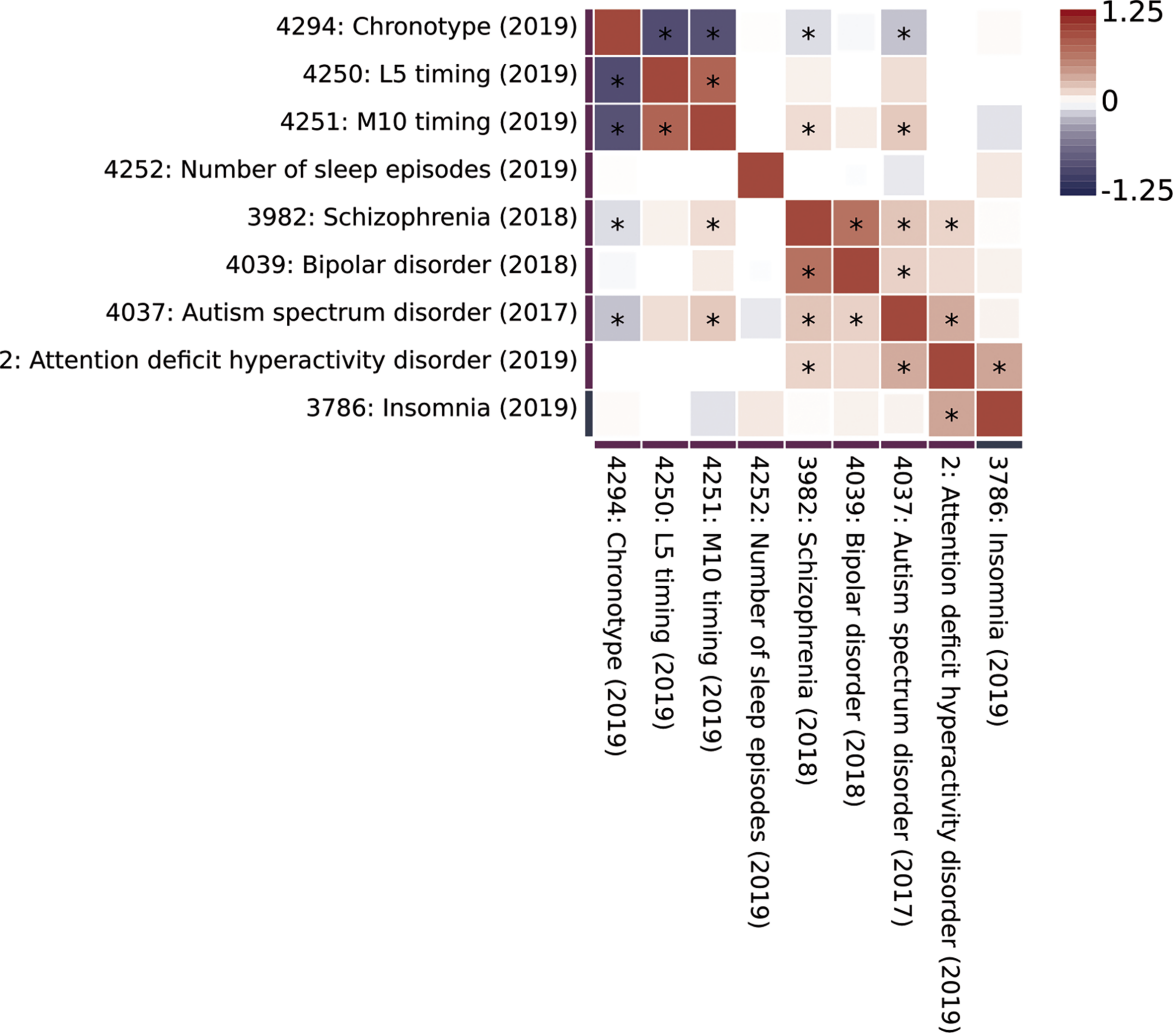
Genetic correlation results indicating shared biology of neuropsychiatric disorders and sleep‐related phenotypes. Symmetric heatplot displaying genetic correlations where those denoted by ‘*’ are significant at *p* < 0.05 after Bonferroni correction for all tests. L5 timing is the midpoint of the least active 5 h of each day. M10 timing is the timing of the most active 10 h of each day. This was generated using genome‐wide association studies (GWAS) data from European ancestry individuals from GWASatlas. GWASatlas ID and year of publication (brackets) are included for each phenotype. Results are clustered by degree of genetic correlation.

Genetic correlation has also been used to show significant genetic overlap between sleep‐related phenotypes and neuropsychiatric disorders. Jones et al^7^ reports that morningness is positively correlated with subjective well‐being and negatively correlated with SZ, depressive symptoms and MDD, providing further evidence of shared biology. Figure 1 shows up‐to‐date results obtained using the GWASatlas repository^38^ of GWAS results. These data firstly indicate strong genetic correlations within the group of neuropsychiatric disorders as well as strong genetic correlations within sleep‐related phenotypes. Across these two groups of phenotypes, morningness was found to be negatively correlated with ASD and SZ but M10 timing, the midpoint of most active 10 hours, was found to be positively correlated with ASD and SZ. Sleep duration was found to be positively correlated with BD and SZ and negatively correlated with insomnia. However, while these correlations are useful for identifying traits that influence one another, they do not show the causal associations between traits.

### 1.4 Mendelian randomization for causal inference using genetic data

#### 1.4.1 What is MR?

MR is a post‐GWAS analysis that can provide evidence to support or reject hypotheses of causal relationships between an exposure trait and risk for a disease outcome.^39^

#### 1.4.2 What are IVs?

Trait‐associated genetic variants, which are strongly associated with an exposure trait, are employed as instrumental variants (IVs) to represent the exposure and assess it as a potentially causal risk factor for the outcome. For polygenic traits, the IVs selected are typically independent SNPs identified through GWAS.

#### 1.4.3 How does MR compare with randomized controlled trials?

**FIGURE 2:**
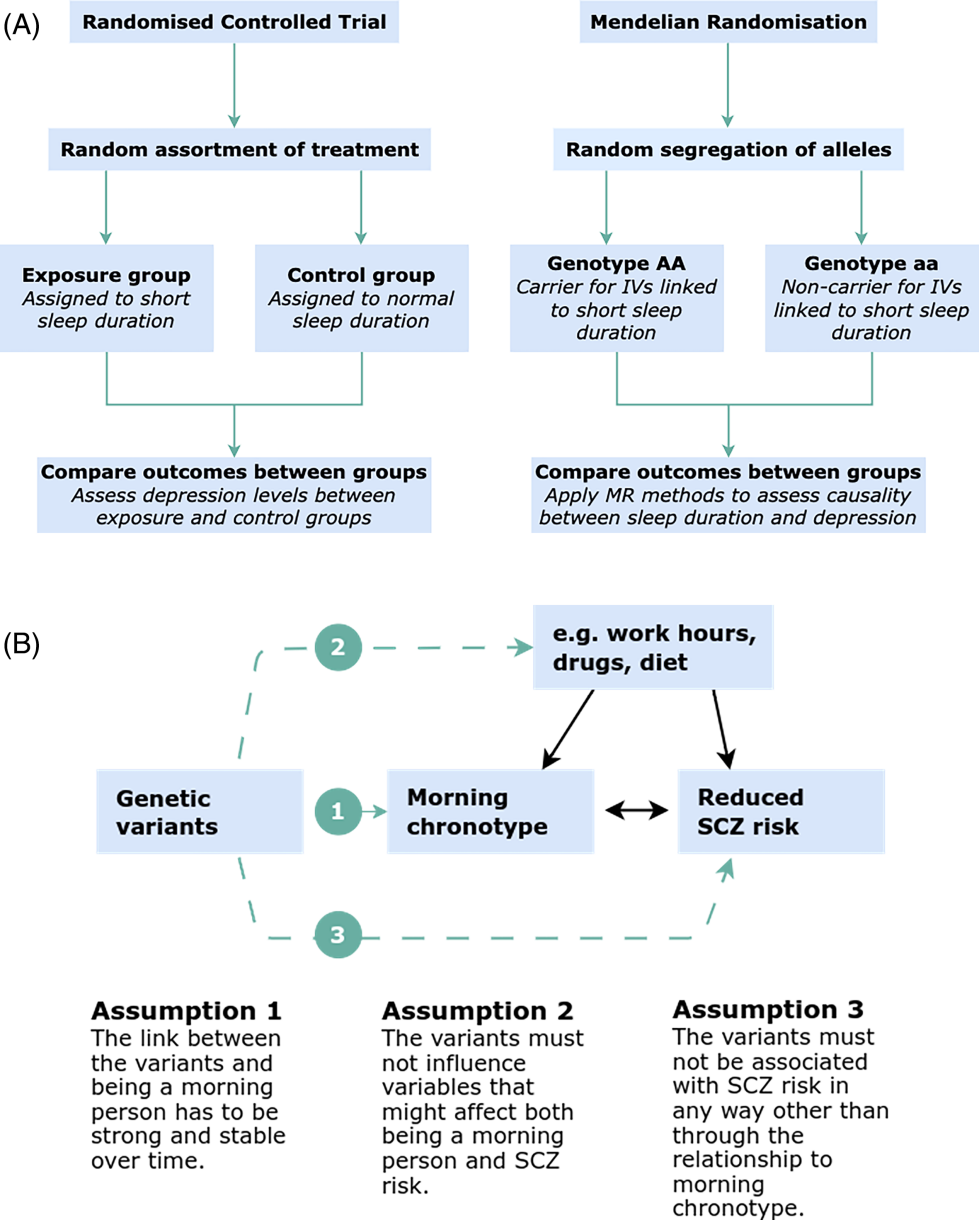
Mendelian randomisation concept and design. (A) Comparison of randomized controlled trial and MR study design. During a randomized controlled trial (RCT), study participants are randomly assorted during study design into two groups and an exposure (e.g., a treatment) is applied to one group to understand the extent to which it influences risk for the outcome. Outcomes (e.g., depression) are compared between both groups and any difference is considered to be due to the exposure treatment. Some treatments such as a set sleep duration are impractical and unethical. Alternatively, MR can be used for any genetically‐based traits. During MR, SNP alleles are assigned as instrumental variants (IVs) to assign groups. One group represents the exposure‐risk allele (e.g. carriers of alleles associated with short sleep duration) and one group with the other non‐risk allele. Outcomes are compared between both groups and any difference is considered to be due to the genetically‐based exposure. (B) Schematic diagram of MR study design and assumptions. During an MR analysis, the effect estimates for genetic instruments are extracted from genome‐wide association studies data and used to approximate the effect of the exposure on the outcome. The study design of MR is dependent on three core assumptions—(1) the genetic instruments are robustly associated with the exposure, (2) genetic instruments have no association with outcome that is not mediated through the risk factor and (3) genetic instruments are not directly associated with the outcome.^40^

The MR concept and design is outlined and compared with randomized controlled trials (RCTs) in Figure 2A. Conditional on some assumptions, MR is similar to RCTs, which are performed to study the effect of various therapies, exposures, or behaviours on disease risk. During an RCT, participants are randomly assigned to one of two study groups, such as an exposure (treatment) group and a control group. The results are compared between the two groups and any statistically significant difference is deemed to be due to the assigned exposure or treatment. In MR analysis, individuals with trait associated alleles are roughly analogous to the study group assigned to treatment in a randomized trial (Figure 2A).^39^ In a true randomized trial, individuals are assigned at random to treatment and control groups. However, MR exploits the random assignment of disease associated alleles (conditional on parental genotype) at conception to individuals to assign individuals to study groups.

### 1.5 IV selection process

IVs are selected based on their known biological function or statistical association with the exposure trait from GWAS and must meet the IV assumptions.^41^ If there is a causal relationship between the exposure and the outcome, the IVs that are associated with the exposure will be associated with the outcome in a proportional way—the effect sizes can be much smaller, but a SNP with a large effect on exposure will have a large effect on outcome, and similarly for small effect.

### 1.6 Assumptions

Figure 2B outlines a pertinent example for an MR investigation of the causal role of the morning chronotype on SZ risk. There are three core IV assumptions that generally must hold in order for the MR study to be valid: (1) IVs are robustly associated with the risk factor, hence usually only SNPs that are independent and GWS are used as IVs; the relevance assumption. (2) IVs share no common causes with the outcome; the independence assumption (sometimes known as the marginal exchangeability assumption). (3) IVs have no association with the outcome that is not mediated through the risk factor; the exclusion restriction assumption.

Potential sources of bias include weak instruments, horizontal pleiotropy and reverse causality.

Violation of the relevance assumption (Assumption 1) can introduce weak instrument bias, a phenomenon causing large statistical variability and bias in the MR estimate when variants only weakly associated with the trait are used as instruments.^42^ The values used to measure instrument strength include R2, a measure that estimates the variance of the trait explained by the variant(s), and the *F*‐statistic, which accounts for R2, sample size and the number of IVs. In Figure 2B, weak instrument bias is introduced if the link between genetic variants and the morning chronotype is not strong, for example, if the variants used as IVs are not GWS for morning chronotype.

Violation of the independence assumption (Assumption 2) can introduce bias through confounders.^43^ Confounding refers to the case where a characteristic influences the exposure and outcome through a ‘backdoor pathway’ between the exposure and outcome.^44^ This can occur when the genetic variants used as IVs influence both the confounders and the outcome, which could exaggerate or attenuate the causal effect estimate. This scenario is analogous to horizontal pleiotropy, whereby an IV influences multiple traits and influences the outcome through an alternative biological pathway that is beyond the examined relationship. Figure 2B illustrates how bias from horizontal pleiotropy can occur if genetic variants linked to the morning chronotype also influence confounders (e.g., work hours, drugs, diet).

Bias from horizontal pleiotropy can also occur through violation of the exclusion restriction assumption (Assumption 3) whereby the genetic variants selected as IVs are associated with the outcome but not through the hypothesized exposure‐outcome pathway. Figure 2B depicts a scenario whereby this assumption is void and IVs for morning chronotype are also linked to SZ. Bias can be investigated by using MR methods developed to detect horizontal pleiotropy such as MR‐Egger,^45^ weighted median^46^ and mode‐based MR.^47^ Table S1 provides definitions from the MR dictionary of each of these MR methods. Beyond the core IV assumptions, the researcher should also examine whether reverse causality is observable. Should valid IVs be available for both the exposure and the outcome, a bidirectional MR should be applied to determine whether the outcome is influencing the exposure.

There are several methodological approaches to MR but two‐sample MR (2SMR) is often the most convenient to apply^48^ and was the method used for all the studies reviewed here. 2SMR uses GWAS summary statistics that are often publicly available and can increase power and improve predictive ability. IVs are selected using the GWAS data for the exposure and then the test statistics for the corresponding IVs are extracted from the outcome GWAS data. There are three additional assumptions for 2SMR analysis: (1) the two GWAS samples are non‐overlapping, (2) the two samples are from the same underlying population and (3) genetic instruments are harmonized between both samples so that the effect or risk allele and the other non‐risk allele are concordant between the two GWAS datasets.^44^

### 1.7 Application of MR to neuropsychiatric disorders and sleep‐related phenotypes

Epidemiological studies of neuropsychiatric disorders have been used to evaluate the contribution of exposures such as genetics, environmental factors and prenatal factors. While epidemiological studies have been useful for understanding the aetiology of neuropsychiatric disorders, it is not ethically possible to perform interventional methods such as RCTs, due to the exposure of participants to potentially harmful risk factors that could initiate or exacerbate negative features of neuropsychiatric disorders. However, due to the increasing number of GWAS studies and genetic correlation studies, it has been possible to identify potential risk factors using MR research to assess causality.

Numerous MR studies have been published that report evidence for associations between neuropsychiatric disorders and sleep‐related phenotypes. These studies used genetically proxied exposures to assess causality for outcomes of interest. Several of these studies used overlapping GWAS datasets (e.g., UK Biobank, Psychiatric Genomics Consortium, 23andMe). However, there is no included study that uses identical MR methodologies, GWAS datasets and IV selection process. All these MR studies used samples of European ancestry. We searched PubMed up to 25th April 2023 for MR studies investigating the association of sleep phenotypes with neuropsychiatric disorders using Mendelian randomization. We used the search term [(Mendelian randomization OR Mendelian randomization) AND (neuropsychiatric OR psychiatric) AND (autism OR ASD OR schizophrenia OR depressive OR depression OR ADHD OR attention deficit OR bipolar OR BD) AND (chronotype OR sleep)]. We also screened the relevant GWAS for each of the included neuropsychiatric disorders for Mendelian randomization analysis performed that investigated the link with sleep or chronotype. We extracted the exposure, outcome, genetic instrument, MR design (one sample or two‐sample and the relevant population) and the type of MR that was performed to investigate the causal effects and the MR sensitivity tests. The search strategy yielded 30 original search results, of which 15 included relevant MR analyses that investigated the causal relationship between neuropsychiatric disorders and sleep traits. Table S2 contains all significant and non‐significant MR analyses and lists the exposure and outcome GWAS data for each.

**FIGURE 3:**
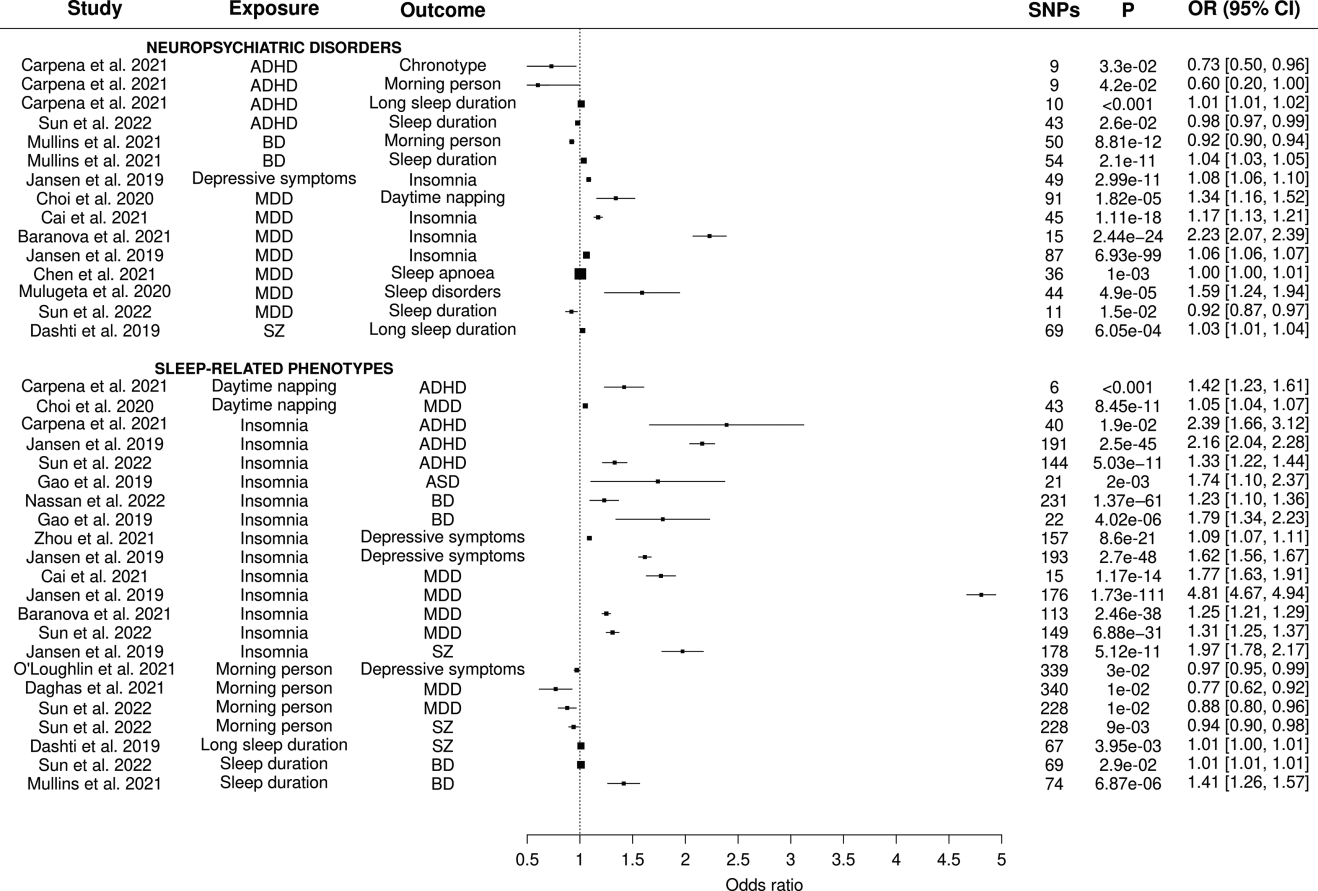
Mendelian randomisation analysis for neuropsychiatric disorders and sleep‐related phenotypes. Results outlined in this figure were compiled from all MR analyses which investigate causal relationships between neuropsychiatric disorders and sleep‐related phenotypes. Bold headers indicate the exposure category. Column ‘SNPs’ indicates the number of genetic instruments used for the exposure. Each point represents the odds ratio and confidence intervals. Where necessary, beta values were converted to odds ratios. The size of each point is proportional to the inverse standard error, meaning that larger points are indicative of more precise estimates. The *p*‐value is as reported in the original study. Odds ratios below 1 indicate that the exposure/risk factor is protective for the outcome, while odds ratios above 1 indicate that the exposure/risk factor increases risk for the outcome.

The methods to address IV assumptions and bias, such as sample overlap and reverse causality, for each of these studies are outlined in Table S3. All studies used IVs identified from GWAS data. Generally, the p‐value threshold of <5 × 10^−8^ was used to assess strength of association; however, the *p*‐value was reduced in several studies to assess reverse causality. The number of IVs used in the MR analyses included in this review ranged from 6 to 340. There were several MR methods used in these studies to infer causality including inverse‐variance weighted (IVW), penalized‐weighted median (PMW), MR‐Egger and MR Pleiotropy RESidual Sum and Outlier (MR‐PRESSO) methods. The results are outlined in Figure 3 and classified according to the type of exposure (neuropsychiatric disorders or sleep‐related phenotypes).

From these 15 studies, 37 gave significant MR results between neuropsychiatric disorders and sleep‐related phenotypes which are interpreted as consistent with a causal relationship. Of these 37 relationships, a total of 15 were identified where a neuropsychiatric disorder was found to be associated with a sleep‐related phenotype and a total of 22 were found where a sleep‐related phenotype was found to be associated with a neuropsychiatric disorder. Figure 3 features results from the MR method that the authors used to conclude as showing evidence for a significant causal relationship. However, each MR method has advantages and limitations and interpretations of a significant result can vary.

#### 1.7.1 Neuropsychiatric disorders as an exposure for sleep‐related phenotypes

The results from MR studies of neuropsychiatric disorders as exposures found that five neuropsychiatric disorders and symptoms (ADHD, BD, depressive symptoms, MDD and SZ) were found to be associated with sleep‐related phenotypes. ADHD was found to be associated with lower probability of being a morning person and causal for long sleep duration.^49^ However, ADHD was conversely reported as associated with reduced sleep duration by Sun et al.^50^

BD was found to be associated with increased sleep duration and lowered probability of being a morning person.^28^ Depressive symptoms were found to be associated with increased insomnia risk.^25^ MDD was found to increase risk for daytime napping,^51^ insomnia,^52^ sleep apnoea^53^ and sleep disorders^54^ and associated with short sleep duration.^49^ MDD was reported as associated with insomnia in three separate studies^25^, ^52^, ^55^, ^56^; however there was wide variation in the reported OR (1.06–2.23) but concordant direction of effect. Finally, SZ was found to be associated with longer sleep duration.^25^

#### 1.7.2 Sleep‐related phenotypes as an exposure for neuropsychiatric disorders

The results from MR studies of sleep‐related phenotypes as exposures found that four sleep‐related phenotypes (daytime napping, insomnia, long sleep duration and sleep duration) were associated with increased risk of neuropsychiatric disorders and morningness was found to be associated with lower risk of neuropsychiatric disorders (Figure 3). Daytime napping was found to be associated with increased risk for ADHD^49^ and MDD.^51^ Carpena et al also report that short sleep duration is associated with ADHD, however this result was not displayed here due to relatively weak evidence of causality with very large estimated effect size across MR methods, and inverse direction of effect across MR methods. Insomnia was found to be associated with increased risk for ADHD,^25^, ^49^, ^50^ ASD,^57^ BD,^57^, ^58^ depressive symptoms^25^, ^59^ and MDD.^25^, ^52^, ^55^, ^56^ Being a morning person was found to be associated with decreased risk of depressive symptoms,^60^ MDD^50^, ^61^ and SZ.^50^ Long sleep duration was found to be associated with increased risk for SZ^32^ and sleep duration was found to increase risk for BD.^28^, ^50^

## 2 DISCUSSION

Here, we have reviewed GWAS for neuropsychiatric disorders and sleep‐related phenotypes with a particular focus on MR to examine the potential causal relationships between neuropsychiatric disorders and sleep‐related phenotypes. GWAS has led to the identification of hundreds of SNPs that are associated with neuropsychiatric disorders and sleep‐related phenotypes. Post‐GWAS analyses using various methods has led to characterization of putative risk genes and biological pathways.

The MR studies outlined in this review have identified 37 potentially causal relationships between neuropsychiatric disorders and sleep‐related phenotypes. All the included MR studies performed numerous MR methodologies to assess causal effect and sensitivity tests to assess bias. Five neuropsychiatric disorders and symptoms (ADHD, BD, depressive symptoms, MDD and SZ) were found to influence risk for sleep‐related phenotypes while five sleep‐related traits (daytime napping, insomnia, morning person, long sleep duration and sleep duration) were found to be associated with neuropsychiatric disorders. Insomnia was found to be associated with ADHD, ASD, BD, depressive symptoms and MDD. Insomnia was identified as having a bidirectional causal relationship with MDD, highlighting the intricate relationship between neuropsychiatric disorders and sleep‐related phenotypes, by which one can exacerbate the other. One other potential bidirectional causal relationship was identified (BD and sleep duration). It was not possible to test for a bidirectional causal relationship between all combinations of phenotypes as some lacked sufficient GWS SNPs as IVs. Therefore, the true nature of the causal relationship between some phenotypes remains to be determined. Those analyses will require further growth of GWAS and an increased number of SNPs that can be used as IVs.

Several potential causal relationships reported in this review were reported in more than one MR study. These studies reported variable effect size estimates but had consistent concordance in the predicted direction of effect, with the caveat that not all studies may have used independent samples. Therefore, it is important to recognize that MR research can be accurate in identifying risk factors and predicting the direction of effect but the effect size is representative of lifetime contribution to risk and does not represent the effect size that an intervention would cause at a specific time.^44^ Exposures that are modifiable risk factors have clear clinical relevance and can influence treatment, if follow‐up studies validate these associations. Other exposures (e.g., SZ) give insight into the biological aetiology of the associated outcomes.

These results provide evidence that sleep and circadian rhythm can contribute to risk for neuropsychiatric disorders, but also for effects occurring in the opposite direction. While neuropsychiatric disorder patients have been observed to experience effects on sleep, it has been difficult to ascertain whether this is a cause or effect of their illness. MR has provided an opportunity to utilize the genetic component of neuropsychiatric disorders, sleep and circadian rhythm to infer causal relationships. Currently, the sleep phenotype with the most power is chronotype. The genetic factor of chronotype allows MR analysis to be performed using this phenotype however given that chronotype is self‐reported, it is not an ideal assessment of circadian rhythm alterations and further studies may integrate data on measurements such as dim‐light melatonin onset,^62^ which give a more accurate representation of the impact of sleep and circadian rhythm alterations on neuropsychiatric disorder risk.^61^

Some areas of concern in the included MR analyses include the decision in some published studies to lower the *p*‐value threshold for IVs, which could potentially void the relevance assumption. A *p*‐value threshold from GWAS of <5 × 10^−8^ should typically be employed at a minimum and *F*‐statistics should also be employed to assess the strength of the instrument. However, with larger GWAS and greater utilization of MR, further evidence for the role of circadian rhythm in neuropsychiatric disorders is expected. Additionally, in some cases, evidence of a causal effect is reported despite lack of consistency in effect estimates across MR and sensitivity tests and, in some cases, inverse trends in the main MR and sensitivity tests.

Variations in MR analysis exist that may enhance studies by reducing bias and integrating multi‐omic data. For example, an approach has been developed to differentiate genetic instruments based on their predicted mechanism.^63^ The approach can then identify when a risk factor is causing multiple biochemical changes that influence risk for an outcome. Implementation of this method could be useful for neuropsychiatric disorders, where many of the GWS loci used as genetic instruments have both protective and causal effects. As many GWS loci can also be eQTLs, the incorporation of expression and gene regulation data in an MR framework can be used to identify causal gene‐trait associations and complement GWAS results and identify priority genes. Transcriptome‐wide summary statistics‐based MR approach (TWMR) uses gene expression changes as an exposure to understand its effect on an outcome and has led to 3913 novel gene‐trait associations, many of which were later found in larger GWAS.^64^ TWMR can incorporate multi‐omic data, such as methylation QTLs, to find functional changes that are causal for an outcome.

In conclusion, GWAS and MR have been useful for understanding the complex, bidirectional relationship between neuropsychiatric disorders and sleep. The phenotypic variance of neuropsychiatric disorders has made it difficult to generate knowledge on the core biology and has hindered the development of effective treatments for these disorders. MR enables a powerful strategy that focuses on understanding the relationship between circadian rhythm and neuropsychiatric disorders without requiring a complete understanding of the underlying biological origins of neuropsychiatric disorders. In this sense, MR is an epidemiological tool that employs genetic data. MR studies can also help us in navigating drug development targets and disproving other pathways that are less likely to be successful therapies. One example is the longitudinal study SELECT that studied the effect of selenium on prostate cancer risk and included 30,000 participants, cost $114 million and lasted 7 years. However, no evidence for a protective effect of selenium on prostate cancer was found.^65^ A subsequent MR analysis replicated these findings and was significantly faster and less expensive.^65^ Similarly in psychiatric research, MR analysis may not only be cost‐effective but also the most viable option for examining causal links. It is important to remain cautious of results from MR. MR replaces an unverifiable set of assumptions (no confounding) with another variable set of assumptions (no pleiotropy, independence), which may be slightly more reasonable than the observational estimate in certain settings. While MR studies may provide evidence for an association or no association, a randomized trial might still be needed to confirm this.

In summary, there is evidence consistent with a causal role for neuropsychiatric disorders (ADHD, BD, MDD and SZ) in sleep‐related phenotypes and evidence that sleep‐related phenotypes are associated with increased risk for neuropsychiatric disorders. These MR results show a bidirectional relationship between neuropsychiatric disorders and sleep‐related phenotypes and identify potential risk factors for follow‐up studies.

## FUNDING INFORMATION

This research was funded by Science Foundation Ireland (SFI) through the SFI Centre for Research Training in Genomics Data Science under grant number 18/CRT/6214 (SC). This publication has emanated from research supported in part by grants from SFI under grant number 15/SIRG/3324 and from the European Research Council (ERC) under the European Union's Horizon 2020 research and innovation program (grant agreement No 950010) (L.M.L.).

## ETHICS STATEMENT

Data were directly downloaded from published studies and no additional ethics approval was needed. Each study is referenced and details on ethics approval are available in each original study.

## Supporting information


**Data S1:** Supporting information.

## Data Availability

Data sharing not applicable to this article as no datasets were generated or analysed during the current study.
